# Methyl 2-{[(3-methyl-5-oxo-1-phenyl-4,5-dihydro-1*H*-pyrazol-4-yl­idene)(thio­phen-2-yl)meth­yl]amino}-3-phenyl­propionate

**DOI:** 10.1107/S1600536811029904

**Published:** 2011-07-30

**Authors:** Hualing Zhu, Xinxin Zhao, Zhan Wang, Junjie Ren, Miao Zhang

**Affiliations:** a Department of Basic Science, Tianjin Agricultural College, Tianjin Jinjing Road No. 22, Tianjin 300384, People’s Republic of China

## Abstract

In the title compound, C_25_H_23_N_3_O_3_S, an intra­molecular N—H⋯O inter­action generates an *S*(6) ring, which stabilizes the enamine–keto form of the compound. This *S*(6) ring and the pyrazole ring are essentially coplanar, making a dihedral angle of 1.49 (6)°. The bond lengths within the *S*(6) ring of the mol­ecule lie between classical single- and double-bond lengths, indicating extensive conjugation. The structure exhibits a thienyl-ring flip disorder, with occupancy factors in the ratio 64.7 (3):35.3 (3).

## Related literature

The high biological activities of pyrazole derivatives are reported by Li *et al.* (2004 ▶) and Tan *et al.*(2009 ▶). The anti­bacterial and biological activities of amino acid esters are described by Xiong *et al.* (1993 ▶). Structures related to the title compound have been reported by Zhu *et al.* (2010 ▶) and Zhang *et al.* (2010 ▶).
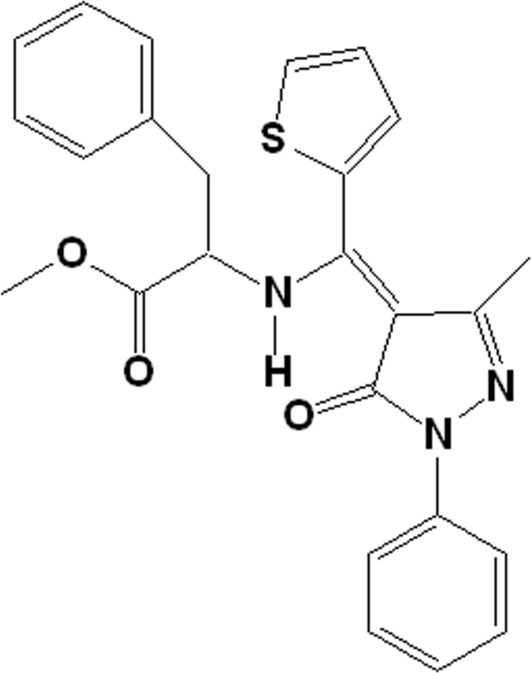


## Experimental

### 

#### Crystal data

C_25_H_23_N_3_O_3_S
*M* *_r_* = 445.52
Monoclinic, 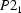

*a* = 6.649 (2) Å
*b* = 18.712 (6) Å
*c* = 9.349 (3) Å
β = 104.903 (5)°
*V* = 1124.0 (7) Å^3^
*Z* = 2
Mo *K*α radiation
μ = 0.18 mm^−1^
*T* = 113 K
0.20 × 0.18 × 0.12 mm

#### Data collection

Rigaku Saturn724 CCD diffractometer
Absorption correction: multi-scan (*CrystalClear*; Rigaku, 2008 ▶) *T*_min_ = 0.966, *T*_max_ = 0.979
11848 measured reflections
2747 independent reflections
2315 reflections with *I* > 2σ(*I*)
*R*_int_ = 0.041

#### Refinement

*R*[*F* ^2^ > 2σ(*F* ^2^)] = 0.034
*wR*(*F* ^2^) = 0.064
*S* = 0.98
2747 reflections
353 parameters
215 restraints
H atoms treated by a mixture of independent and constrained refinement
Δρ_max_ = 0.15 e Å^−3^
Δρ_min_ = −0.20 e Å^−3^
Absolute structure: Flack (1983 ▶), 2401 Friedel pairs
Flack parameter: 0.05 (8)

### 

Data collection: *CrystalClear* (Rigaku, 2008 ▶); cell refinement: *CrystalClear*; data reduction: *CrystalClear*; program(s) used to solve structure: *SHELXS97* (Sheldrick, 2008 ▶); program(s) used to refine structure: *SHELXL97* (Sheldrick, 2008 ▶); molecular graphics: *ORTEP-3* (Farrugia, 1997 ▶); software used to prepare material for publication: *SHELXL97*.

## Supplementary Material

Crystal structure: contains datablock(s) I, global. DOI: 10.1107/S1600536811029904/om2448sup1.cif

Structure factors: contains datablock(s) I. DOI: 10.1107/S1600536811029904/om2448Isup2.hkl

Supplementary material file. DOI: 10.1107/S1600536811029904/om2448Isup3.cml

Additional supplementary materials:  crystallographic information; 3D view; checkCIF report

## Figures and Tables

**Table 1:** Hydrogen-bond geometry (Å, °)

*D*—H⋯*A*	*D*—H	H⋯*A*	*D*⋯*A*	*D*—H⋯*A*
N3—H1⋯O1	0.88	1.93	2.668 (2)	141

**Fig. 1.**
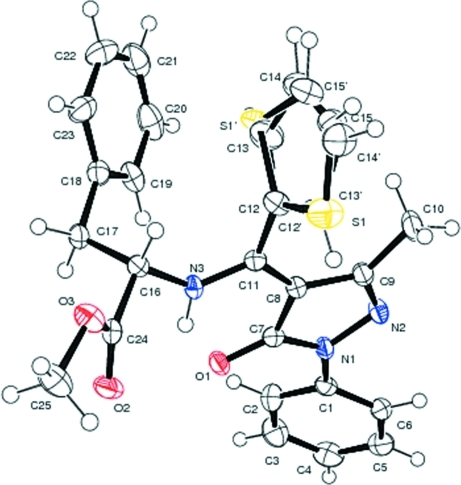
The molecular structure of the title compound with the atom numbering scheme. Displacement ellipsoids are drawn at the 30% probability level. H atoms are presented as a small spheres of arbitrary radii.

## supplementary crystallographic information

### Comment

Pyrazole derivatives have drawn attentionfrom agricultural chemists for their high biological activity and low toxicity. They are widely used as pesticide, miticide and weed killers, and with the positional changes of the substituent group of pyrazole ring, more and more new pyrazole agricultural chemicals are synthesized and commercialized (Tan *et al.*,2009), so pyrazole derivatives have become one of the focal points to the creation of new agricultural chemicals. Amino acid esters also possess good antibacterial and biological activity (Xiong *et al.*,1993).

In the molecule of the title compound (Fig. 1), there is an intramolecular N3—H1···O1 interaction that generates a S(6) ring, and stabilizes the enamine–keto form of the compound. The dihedral angle between this S(6) ring and the pyrazole ring is 1.49 (6)°, indicating that they are essentially coplanar, as seen in Methyl 2-{[(1*Z*)-(3-methyl-5-oxo-1-phenyl-4,5-dihydro-1*H*-pyrazol-4- ylidene)(methyl)methyl]amino}-3-phenylpropanoate(1.50 (15)°; Zhu *et al.*, 2010). The bond lengths within this part of the molecular lie between classical single-and double-bond lengths, indicating extensive conjugation. The S(6) ring makes dihedral angles of 54.29 (6)°,82.21 (22)° and 28.53 (6)° with the benzene ring of phenylalanine methyl ester, the thiazole ring and benzene ring bonded to pyrazole ring, respectively.

Atoms N3, C16, C24 and O2 are not coplanar, the torsion angle is 37.17 (22)°, similar to some other 4-acylpyrazolone Schiff Bases (Zhang *et al.*, 2010). The bond lengths in this part of the molecule indicate that only C24—O2 is a classical double bond, other bonds are classical single bonds.

The structure exhibits a thienyl-ring flip disorder with the occupancy factors in the ratio 67/33.

### Experimental

The title compound was synthesized by refluxing the mixture of 1-phenyl-3-methyl-4-(2-thenoyl)pyrazolone-5 (HPMTP) (10*m* mol) and phenylalanine methyl ester(10*m* mol) in ethanol (100 ml) over a steam bath for about 7 h, then the solution was cooled down to room temperature. After five days, pale yellow blocks were obtained and dried in the air. The product was recrystallized from ethanol which afforded pale yellow crystals suitable for *X*-ray analysis.

### Refinement

The disorder model of thiazole ring was refined using the tools available in *SHELXL97* (Sheldrick, 2008): *DFIX* for restraining distances, FLAT for constraining the thienyl rings to be planar, SIMU for restraining the same *U^ij^* and ISOR for restraining atoms to be approximately isotropic.

All H atoms were geometrically positioned and treated as riding on their parent atoms, with C—H = 0.93 Å for the aromatic, 0.96 Å for the methyl and N—H= 0.88 Å with *U*\ĩso\~(H)= 1.2 *U*\~eq\~C(aromatic, N) or, 1.5*U*\~eq\~C(methyl).

### Crystal data

**Table:**

C_25_H_23_N_3_O_3_S	*F*(000) = 468
*M**_r_* = 445.52	*D*_x_ = 1.316 Mg m^−^^3^
Monoclinic, *P*2_1_	Mo *K*α radiation, λ = 0.71073 Å
*a* = 6.649 (2) Å	Cell parameters from 5148 reflections
*b* = 18.712 (6) Å	θ = 2.2–27.9°
*c* = 9.349 (3) Å	µ = 0.18 mm^−^^1^
β = 104.903 (5)°	*T* = 113 K
*V* = 1124.0 (7) Å^3^	Block, pale yellow
*Z* = 2	0.20 × 0.18 × 0.12 mm

### Data collection

**Table:**

Rigaku Saturn724 CCD diffractometer	2747 independent reflections
Radiation source: rotating anode	2315 reflections with *I* > 2σ(*I*)
graphite	*R*_int_ = 0.041
ω and φ scans	θ_max_ = 27.9°, θ_min_ = 2.2°
Absorption correction: multi-scan (*CrystalClear*; Rigaku, 2008)	*h* = −8→8
*T*_min_ = 0.966, *T*_max_ = 0.979	*k* = −24→24
11848 measured reflections	*l* = −12→12

### Refinement

**Table:**

Refinement on *F*^2^	Secondary atom site location: difference Fourier map
Least-squares matrix: full	Hydrogen site location: inferred from neighbouring sites
*R*[*F*^2^ > 2σ(*F*^2^)] = 0.034	H atoms treated by a mixture of independent and constrained refinement
*wR*(*F*^2^) = 0.064	*w* = 1/[σ^2^(*F*_o_^2^) + (0.0327*P*)^2^] where *P* = (*F*_o_^2^ + 2*F*_c_^2^)/3
*S* = 0.98	(Δ/σ)_max_ < 0.001
2747 reflections	Δρ_max_ = 0.15 e Å^−^^3^
353 parameters	Δρ_min_ = −0.20 e Å^−^^3^
215 restraints	Absolute structure: Flack (1983), 2401 Friedel pairs
Primary atom site location: structure-invariant direct methods	Flack parameter: 0.05 (8)

### Special details

**Table:**

Geometry. All e.s.d.'s (except the e.s.d. in the dihedral angle between two l.s. planes) are estimated using the full covariance matrix. The cell e.s.d.'s are taken into account individually in the estimation of e.s.d.'s in distances, angles and torsion angles; correlations between e.s.d.'s in cell parameters are only used when they are defined by crystal symmetry. An approximate (isotropic) treatment of cell e.s.d.'s is used for estimating e.s.d.'s involving l.s. planes.
Refinement. Refinement of *F*^2^ against ALL reflections. The weighted *R*-factor *wR* and goodness of fit *S* are based on *F*^2^, conventional *R*-factors *R* are based on *F*, with *F* set to zero for negative *F*^2^. The threshold expression of *F*^2^ > σ(*F*^2^) is used only for calculating *R*-factors(gt) *etc*. and is not relevant to the choice of reflections for refinement. *R*-factors based on *F*^2^ are statistically about twice as large as those based on *F*, and *R*- factors based on ALL data will be even larger.

### Fractional atomic coordinates and isotropic or equivalent isotropic displacement parameters (Å^2^)

**Table:**

	*x*	*y*	*z*	*U*_iso_*/*U*_eq_	Occ. (<1)
O1	1.1534 (2)	0.63768 (7)	0.18356 (15)	0.0277 (3)	
O2	0.5930 (2)	0.61573 (8)	−0.05979 (16)	0.0351 (4)	
O3	0.3142 (2)	0.68792 (8)	−0.10026 (16)	0.0306 (4)	
N1	1.3400 (2)	0.65769 (9)	0.42905 (17)	0.0222 (4)	
N2	1.3238 (2)	0.70218 (10)	0.54712 (17)	0.0249 (4)	
N3	0.8186 (3)	0.72194 (9)	0.11372 (18)	0.0247 (4)	
H1	0.8944	0.6858	0.0974	0.028 (6)*	
C1	1.5151 (3)	0.61245 (11)	0.4456 (2)	0.0226 (4)	
C2	1.5855 (3)	0.59401 (13)	0.3226 (2)	0.0314 (5)	
H2	1.5161	0.6115	0.2273	0.038*	
C3	1.7570 (3)	0.55012 (14)	0.3403 (3)	0.0380 (6)	
H3	1.8053	0.5378	0.2564	0.046*	
C4	1.8595 (3)	0.52384 (13)	0.4775 (3)	0.0361 (6)	
H4	1.9765	0.4933	0.4879	0.043*	
C5	1.7900 (3)	0.54243 (12)	0.5997 (3)	0.0317 (5)	
H5	1.8598	0.5246	0.6946	0.038*	
C6	1.6182 (3)	0.58712 (11)	0.5842 (2)	0.0260 (5)	
H6	1.5719	0.6002	0.6686	0.031*	
C7	1.1799 (3)	0.66940 (11)	0.3045 (2)	0.0230 (4)	
C8	1.0560 (3)	0.72515 (10)	0.3459 (2)	0.0209 (4)	
C9	1.1586 (3)	0.74221 (11)	0.4976 (2)	0.0237 (4)	
C10	1.1023 (3)	0.79648 (13)	0.5971 (2)	0.0335 (5)	
H10A	1.2064	0.7961	0.6928	0.050*	
H10B	0.9652	0.7851	0.6119	0.050*	
H10C	1.0983	0.8439	0.5521	0.050*	
C11	0.8795 (3)	0.75220 (10)	0.2467 (2)	0.0209 (4)	
C12	0.7620 (11)	0.8131 (3)	0.2843 (9)	0.0201 (13)	0.647 (3)
C13	0.7922 (16)	0.8830 (4)	0.2580 (9)	0.038 (2)	0.647 (3)
C14	0.6536 (12)	0.9317 (5)	0.3156 (10)	0.0313 (13)	0.647 (3)
C15	0.5342 (14)	0.8917 (3)	0.3807 (9)	0.0291 (14)	0.647 (3)
S1	0.5747 (3)	0.80143 (13)	0.3760 (3)	0.0387 (4)	0.647 (3)
C12'	0.757 (2)	0.8125 (5)	0.2850 (18)	0.028 (2)	0.353 (3)
C13'	0.617 (2)	0.8021 (10)	0.3677 (17)	0.036 (3)	0.353 (3)
H13A	0.5751	0.7559	0.3975	0.043*	0.353 (3)
C15'	0.621 (2)	0.9239 (10)	0.3287 (18)	0.034 (2)	0.353 (3)
C14'	0.528 (3)	0.8741 (7)	0.395 (2)	0.037 (2)	0.353 (3)
S1'	0.7937 (7)	0.8971 (2)	0.2379 (5)	0.0324 (8)	0.353 (3)
C16	0.6425 (3)	0.74125 (11)	−0.0075 (2)	0.0231 (4)	
H16	0.5549	0.7779	0.0258	0.028*	
C17	0.7166 (3)	0.76982 (11)	−0.1407 (2)	0.0255 (5)	
H17A	0.8063	0.7335	−0.1703	0.031*	
H17B	0.5940	0.7775	−0.2254	0.031*	
C18	0.8361 (3)	0.83898 (11)	−0.1058 (2)	0.0270 (5)	
C19	1.0459 (3)	0.83866 (14)	−0.0317 (2)	0.0329 (5)	
H19	1.1163	0.7946	−0.0048	0.040*	
C20	1.1537 (4)	0.90311 (16)	0.0032 (3)	0.0435 (6)	
H20	1.2974	0.9027	0.0535	0.052*	
C21	1.0520 (5)	0.96732 (15)	−0.0351 (3)	0.0496 (7)	
H21	1.1249	1.0111	−0.0100	0.060*	
C22	0.8446 (5)	0.96765 (14)	−0.1097 (3)	0.0512 (7)	
H22	0.7747	1.0118	−0.1372	0.061*	
C23	0.7374 (4)	0.90402 (13)	−0.1449 (2)	0.0386 (6)	
H23	0.5943	0.9049	−0.1966	0.046*	
C24	0.5168 (3)	0.67377 (11)	−0.0565 (2)	0.0246 (5)	
C25	0.1802 (3)	0.62806 (13)	−0.1612 (3)	0.0384 (6)	
H25A	0.2104	0.6123	−0.2534	0.058*	
H25B	0.0343	0.6429	−0.1814	0.058*	
H25C	0.2055	0.5886	−0.0899	0.058*	
H13	0.894 (5)	0.907 (2)	0.219 (4)	0.046*	0.647 (3)
H14	0.639 (10)	0.9824 (9)	0.298 (6)	0.046*	0.647 (3)
H15	0.416 (4)	0.904 (3)	0.416 (4)	0.046*	0.647 (3)
H15'	0.627 (19)	0.9752 (12)	0.327 (11)	0.046*	0.353 (3)
H14'	0.449 (10)	0.892 (4)	0.460 (6)	0.046*	0.353 (3)

### Atomic displacement parameters (Å^2^)

**Table:**

	*U*^11^	*U*^22^	*U*^33^	*U*^12^	*U*^13^	*U*^23^
O1	0.0368 (8)	0.0247 (8)	0.0186 (7)	0.0067 (6)	0.0015 (6)	−0.0056 (6)
O2	0.0449 (9)	0.0197 (8)	0.0349 (9)	0.0023 (8)	−0.0003 (7)	−0.0039 (7)
O3	0.0288 (8)	0.0279 (9)	0.0349 (8)	−0.0047 (7)	0.0078 (6)	−0.0087 (7)
N1	0.0266 (9)	0.0233 (9)	0.0155 (8)	0.0027 (7)	0.0033 (7)	−0.0024 (7)
N2	0.0287 (9)	0.0279 (9)	0.0179 (9)	0.0009 (8)	0.0057 (7)	−0.0056 (7)
N3	0.0332 (10)	0.0204 (9)	0.0174 (8)	0.0069 (8)	0.0007 (7)	−0.0025 (7)
C1	0.0232 (10)	0.0190 (10)	0.0255 (11)	−0.0015 (9)	0.0061 (8)	−0.0012 (8)
C2	0.0318 (12)	0.0378 (13)	0.0256 (11)	0.0017 (10)	0.0093 (9)	0.0003 (10)
C3	0.0329 (13)	0.0452 (14)	0.0379 (13)	0.0052 (11)	0.0127 (10)	−0.0081 (11)
C4	0.0264 (12)	0.0326 (13)	0.0473 (14)	0.0058 (10)	0.0061 (11)	−0.0046 (11)
C5	0.0279 (12)	0.0279 (12)	0.0341 (12)	−0.0020 (10)	−0.0015 (10)	0.0020 (10)
C6	0.0291 (11)	0.0243 (11)	0.0232 (10)	−0.0020 (9)	0.0042 (9)	−0.0010 (9)
C7	0.0280 (11)	0.0183 (10)	0.0216 (10)	−0.0012 (9)	0.0041 (9)	−0.0004 (8)
C8	0.0230 (10)	0.0206 (10)	0.0188 (10)	−0.0010 (9)	0.0047 (8)	−0.0012 (8)
C9	0.0267 (11)	0.0244 (11)	0.0194 (10)	0.0004 (9)	0.0051 (8)	−0.0025 (8)
C10	0.0385 (13)	0.0410 (13)	0.0187 (10)	0.0095 (11)	0.0032 (9)	−0.0069 (10)
C11	0.0264 (11)	0.0173 (10)	0.0196 (10)	−0.0025 (8)	0.0069 (9)	−0.0011 (8)
C12	0.020 (2)	0.022 (2)	0.016 (2)	−0.001 (2)	0.0006 (19)	−0.003 (2)
C13	0.041 (3)	0.031 (4)	0.045 (3)	0.007 (3)	0.016 (2)	0.000 (3)
C14	0.034 (3)	0.021 (2)	0.038 (2)	−0.006 (2)	0.0089 (18)	−0.0018 (19)
C15	0.033 (2)	0.024 (3)	0.035 (2)	0.005 (2)	0.0174 (19)	−0.002 (2)
S1	0.0402 (9)	0.0331 (7)	0.0529 (8)	0.0029 (7)	0.0304 (7)	0.0005 (6)
C12'	0.029 (4)	0.025 (4)	0.031 (4)	0.001 (4)	0.009 (4)	−0.004 (4)
C13'	0.036 (4)	0.031 (4)	0.041 (4)	0.011 (4)	0.013 (4)	−0.011 (3)
C15'	0.038 (4)	0.022 (4)	0.043 (4)	0.000 (4)	0.012 (3)	−0.007 (3)
C14'	0.037 (3)	0.037 (4)	0.043 (4)	−0.002 (4)	0.018 (3)	0.001 (4)
S1'	0.0388 (13)	0.0156 (12)	0.0468 (15)	−0.0019 (10)	0.0183 (11)	−0.0063 (11)
C16	0.0286 (11)	0.0199 (10)	0.0186 (10)	0.0026 (9)	0.0020 (9)	−0.0013 (8)
C17	0.0310 (12)	0.0252 (11)	0.0169 (10)	0.0014 (9)	0.0000 (9)	−0.0017 (8)
C18	0.0330 (12)	0.0277 (12)	0.0206 (10)	−0.0031 (10)	0.0073 (9)	−0.0021 (9)
C19	0.0322 (13)	0.0415 (14)	0.0262 (11)	−0.0043 (11)	0.0095 (10)	−0.0095 (11)
C20	0.0389 (14)	0.0619 (18)	0.0340 (13)	−0.0207 (14)	0.0174 (11)	−0.0165 (13)
C21	0.078 (2)	0.0411 (16)	0.0351 (13)	−0.0313 (15)	0.0243 (14)	−0.0092 (12)
C22	0.079 (2)	0.0269 (13)	0.0446 (15)	−0.0080 (14)	0.0107 (15)	0.0088 (11)
C23	0.0482 (14)	0.0307 (12)	0.0322 (12)	−0.0045 (11)	0.0019 (11)	0.0068 (11)
C24	0.0343 (12)	0.0234 (11)	0.0156 (10)	−0.0001 (10)	0.0056 (9)	0.0001 (8)
C25	0.0351 (13)	0.0377 (14)	0.0431 (14)	−0.0114 (11)	0.0112 (11)	−0.0139 (11)

### Geometric parameters (Å, °)

**Table:**

O1—C7	1.249 (2)	C14—H15'	0.85 (5)
O2—C24	1.202 (2)	C15—S1	1.712 (5)
O3—C24	1.330 (2)	C15—H15	0.959 (10)
O3—C25	1.453 (2)	C15—H14'	1.04 (4)
N1—C7	1.378 (2)	S1—H13A	0.8749
N1—N2	1.409 (2)	C12'—C13'	1.368 (9)
N1—C1	1.415 (3)	C12'—S1'	1.678 (9)
N2—C9	1.311 (3)	C13'—C14'	1.518 (10)
N3—C11	1.331 (2)	C13'—H13A	0.9699
N3—C16	1.450 (3)	C15'—C14'	1.349 (9)
N3—H1	0.8800	C15'—S1'	1.674 (9)
C1—C6	1.384 (3)	C15'—H14	1.15 (3)
C1—C2	1.392 (3)	C15'—H15'	0.962 (11)
C2—C3	1.380 (3)	C14'—H15	1.00 (3)
C2—H2	0.9500	C14'—H14'	0.961 (11)
C3—C4	1.379 (3)	S1'—H13	0.76 (2)
C3—H3	0.9500	C16—C24	1.519 (3)
C4—C5	1.382 (3)	C16—C17	1.547 (3)
C4—H4	0.9500	C16—H16	1.0000
C5—C6	1.393 (3)	C17—C18	1.510 (3)
C5—H5	0.9500	C17—H17A	0.9900
C6—H6	0.9500	C17—H17B	0.9900
C7—C8	1.443 (3)	C18—C19	1.389 (3)
C8—C11	1.391 (3)	C18—C23	1.386 (3)
C8—C9	1.442 (3)	C19—C20	1.398 (4)
C9—C10	1.489 (3)	C19—H19	0.9500
C10—H10A	0.9800	C20—C21	1.380 (4)
C10—H10B	0.9800	C20—H20	0.9500
C10—H10C	0.9800	C21—C22	1.376 (4)
C11—C12	1.474 (5)	C21—H21	0.9500
C11—C12'	1.489 (9)	C22—C23	1.383 (3)
C12—C13	1.356 (7)	C22—H22	0.9500
C12—S1	1.698 (5)	C23—H23	0.9500
C13—C14	1.491 (8)	C25—H25A	0.9800
C13—H13	0.963 (10)	C25—H25B	0.9800
C14—C15	1.345 (6)	C25—H25C	0.9800
C14—H14	0.963 (10)		
			
C24—O3—C25	115.95 (17)	H15—C15—H14'	27 (5)
C7—N1—N2	111.72 (15)	C12—S1—C15	91.5 (4)
C7—N1—C1	128.47 (17)	C12—S1—H13A	106.4
N2—N1—C1	119.63 (15)	C15—S1—H13A	161.0
C9—N2—N1	106.65 (15)	C13'—C12'—C11	121.6 (10)
C11—N3—C16	127.91 (17)	C13'—C12'—S1'	116.7 (10)
C11—N3—H1	116.0	C11—C12'—S1'	121.6 (7)
C16—N3—H1	116.0	C12'—C13'—C14'	108.5 (15)
C6—C1—C2	119.8 (2)	C12'—C13'—H13A	125.1
C6—C1—N1	120.16 (18)	C14'—C13'—H13A	126.3
C2—C1—N1	120.00 (18)	C14'—C15'—S1'	118.6 (15)
C3—C2—C1	119.4 (2)	C14'—C15'—H14	150 (3)
C3—C2—H2	120.3	S1'—C15'—H14	92 (3)
C1—C2—H2	120.3	C14'—C15'—H15'	137 (6)
C4—C3—C2	121.3 (2)	S1'—C15'—H15'	104 (7)
C4—C3—H3	119.3	H14—C15'—H15'	15 (8)
C2—C3—H3	119.3	C15'—C14'—C13'	107.1 (18)
C3—C4—C5	119.2 (2)	C15'—C14'—H15	99 (3)
C3—C4—H4	120.4	C13'—C14'—H15	151 (3)
C5—C4—H4	120.4	C15'—C14'—H14'	116 (6)
C4—C5—C6	120.3 (2)	C13'—C14'—H14'	136 (5)
C4—C5—H5	119.9	H15—C14'—H14'	28 (5)
C6—C5—H5	119.9	C15'—S1'—C12'	89.0 (8)
C1—C6—C5	119.9 (2)	C15'—S1'—H13	144 (3)
C1—C6—H6	120.1	C12'—S1'—H13	119 (3)
C5—C6—H6	120.1	N3—C16—C24	107.42 (16)
O1—C7—N1	126.01 (18)	N3—C16—C17	110.79 (17)
O1—C7—C8	129.01 (18)	C24—C16—C17	108.01 (16)
N1—C7—C8	104.97 (16)	N3—C16—H16	110.2
C11—C8—C9	132.86 (18)	C24—C16—H16	110.2
C11—C8—C7	121.75 (18)	C17—C16—H16	110.2
C9—C8—C7	105.39 (16)	C18—C17—C16	112.39 (16)
N2—C9—C8	111.26 (17)	C18—C17—H17A	109.1
N2—C9—C10	119.42 (18)	C16—C17—H17A	109.1
C8—C9—C10	129.32 (18)	C18—C17—H17B	109.1
C9—C10—H10A	109.5	C16—C17—H17B	109.1
C9—C10—H10B	109.5	H17A—C17—H17B	107.9
H10A—C10—H10B	109.5	C19—C18—C23	118.8 (2)
C9—C10—H10C	109.5	C19—C18—C17	120.6 (2)
H10A—C10—H10C	109.5	C23—C18—C17	120.54 (19)
H10B—C10—H10C	109.5	C18—C19—C20	120.1 (2)
N3—C11—C8	118.04 (17)	C18—C19—H19	119.9
N3—C11—C12	119.9 (4)	C20—C19—H19	119.9
C8—C11—C12	122.0 (4)	C21—C20—C19	120.2 (2)
N3—C11—C12'	119.5 (7)	C21—C20—H20	119.9
C8—C11—C12'	122.4 (7)	C19—C20—H20	119.9
C12—C11—C12'	1.4 (7)	C22—C21—C20	119.7 (2)
C13—C12—C11	126.2 (6)	C22—C21—H21	120.1
C13—C12—S1	112.2 (6)	C20—C21—H21	120.1
C11—C12—S1	121.5 (4)	C21—C22—C23	120.3 (3)
C12—C13—C14	112.9 (9)	C21—C22—H22	119.8
C12—C13—H13	132 (3)	C23—C22—H22	119.8
C14—C13—H13	115 (3)	C22—C23—C18	120.8 (2)
C15—C14—C13	108.4 (9)	C22—C23—H23	119.6
C15—C14—H14	125 (3)	C18—C23—H23	119.6
C13—C14—H14	126 (3)	O2—C24—O3	124.90 (19)
C15—C14—H15'	108 (7)	O2—C24—C16	123.79 (19)
C13—C14—H15'	143 (7)	O3—C24—C16	111.26 (17)
H14—C14—H15'	19 (8)	O3—C25—H25A	109.5
C14—C15—S1	115.0 (7)	O3—C25—H25B	109.5
C14—C15—H15	131 (3)	H25A—C25—H25B	109.5
S1—C15—H15	114 (3)	O3—C25—H25C	109.5
C14—C15—H14'	144 (5)	H25A—C25—H25C	109.5
S1—C15—H14'	98 (5)	H25B—C25—H25C	109.5
			
C7—N1—N2—C9	−1.1 (2)	C12'—C11—C12—S1	21 (39)
C1—N1—N2—C9	174.54 (17)	C11—C12—C13—C14	−177.4 (9)
C7—N1—C1—C6	−156.68 (19)	S1—C12—C13—C14	−0.2 (3)
N2—N1—C1—C6	28.5 (3)	C12—C13—C14—C15	0.4 (5)
C7—N1—C1—C2	24.1 (3)	C13—C14—C15—S1	−0.5 (6)
N2—N1—C1—C2	−150.69 (19)	C13—C12—S1—C15	−0.1 (2)
C6—C1—C2—C3	0.5 (3)	C11—C12—S1—C15	177.3 (7)
N1—C1—C2—C3	179.7 (2)	C14—C15—S1—C12	0.4 (4)
C1—C2—C3—C4	0.3 (4)	N3—C11—C12'—C13'	99.4 (8)
C2—C3—C4—C5	−0.6 (4)	C8—C11—C12'—C13'	−79.7 (9)
C3—C4—C5—C6	0.1 (3)	C12—C11—C12'—C13'	−154 (40)
C2—C1—C6—C5	−1.0 (3)	N3—C11—C12'—S1'	−84.4 (11)
N1—C1—C6—C5	179.79 (18)	C8—C11—C12'—S1'	96.5 (11)
C4—C5—C6—C1	0.7 (3)	C12—C11—C12'—S1'	22 (38)
N2—N1—C7—O1	−179.70 (19)	C11—C12'—C13'—C14'	176.6 (17)
C1—N1—C7—O1	5.2 (3)	S1'—C12'—C13'—C14'	0.2 (4)
N2—N1—C7—C8	0.5 (2)	S1'—C15'—C14'—C13'	0.8 (10)
C1—N1—C7—C8	−174.58 (18)	C12'—C13'—C14'—C15'	−0.6 (7)
O1—C7—C8—C11	−0.5 (3)	C14'—C15'—S1'—C12'	−0.6 (7)
N1—C7—C8—C11	179.22 (18)	C13'—C12'—S1'—C15'	0.2 (3)
O1—C7—C8—C9	−179.6 (2)	C11—C12'—S1'—C15'	−176.2 (16)
N1—C7—C8—C9	0.1 (2)	C11—N3—C16—C24	−128.0 (2)
N1—N2—C9—C8	1.1 (2)	C11—N3—C16—C17	114.3 (2)
N1—N2—C9—C10	−178.75 (18)	N3—C16—C17—C18	−65.0 (2)
C11—C8—C9—N2	−179.8 (2)	C24—C16—C17—C18	177.56 (17)
C7—C8—C9—N2	−0.8 (2)	C16—C17—C18—C19	82.1 (2)
C11—C8—C9—C10	0.1 (4)	C16—C17—C18—C23	−96.9 (2)
C7—C8—C9—C10	179.0 (2)	C23—C18—C19—C20	0.5 (3)
C16—N3—C11—C8	−179.25 (18)	C17—C18—C19—C20	−178.42 (19)
C16—N3—C11—C12	0.0 (4)	C18—C19—C20—C21	0.3 (3)
C16—N3—C11—C12'	1.6 (6)	C19—C20—C21—C22	−0.9 (4)
C9—C8—C11—N3	−176.8 (2)	C20—C21—C22—C23	0.7 (4)
C7—C8—C11—N3	4.4 (3)	C21—C22—C23—C18	0.1 (4)
C9—C8—C11—C12	3.9 (4)	C19—C18—C23—C22	−0.7 (3)
C7—C8—C11—C12	−174.9 (3)	C17—C18—C23—C22	178.2 (2)
C9—C8—C11—C12'	2.3 (6)	C25—O3—C24—O2	−2.4 (3)
C7—C8—C11—C12'	−176.5 (5)	C25—O3—C24—C16	175.07 (17)
N3—C11—C12—C13	−87.5 (5)	N3—C16—C24—O2	−37.1 (3)
C8—C11—C12—C13	91.7 (5)	C17—C16—C24—O2	82.4 (2)
C12'—C11—C12—C13	−162 (40)	N3—C16—C24—O3	145.40 (16)
N3—C11—C12—S1	95.4 (5)	C17—C16—C24—O3	−95.05 (19)
C8—C11—C12—S1	−85.4 (5)		

### Hydrogen-bond geometry (Å, °)

**Table:**

*D*—H···*A*	*D*—H	H···*A*	*D*···*A*	*D*—H···*A*
N3—H1···O1	0.88	1.93	2.668 (2)	141.
